# Advances in the Approaches Using Peripheral Perfusion for Monitoring Hemodynamic Status

**DOI:** 10.3389/fmed.2020.614326

**Published:** 2020-12-07

**Authors:** Julianne M. Falotico, Koichiro Shinozaki, Kota Saeki, Lance B. Becker

**Affiliations:** ^1^Department of Emergency Medicine, North Shore University Hospital, Northwell Health, Manhasset, NY, United States; ^2^The Feinstein Institutes for Medical Research, Northwell Health, Manhasset, NY, United States; ^3^Nihon Kohden Innovation Center, Cambridge, MA, United States

**Keywords:** capillary refill time, shock, sepsis, medical device, peripheral perfusion, optics, monitoring, hemodynamic status

## Abstract

Measures of peripheral perfusion can be used to assess the hemodynamic status of critically ill patients. By monitoring peripheral perfusion status, clinicians can promptly initiate life-saving therapy and reduce the likelihood of shock-associated death. Historically, abnormal perfusion has been indicated by the observation of pale, cold, and clammy skin with increased capillary refill time. The utility of these assessments has been debated given that clinicians may vary in their clinical interpretation of body temperature and refill time. Considering these constraints, current sepsis bundles suggest the need to revise resuscitation guidelines. New technologies have been developed to calculate capillary refill time in the hopes of identifying a new gold standard for clinical care. These devices measure either light reflected at the surface of the fingertip (reflected light), or light transmitted through the inside of the fingertip (transmitted light). These new technologies may enable clinicians to monitor peripheral perfusion status more accurately and may increase the potential for ubiquitous hemodynamic monitoring across different clinical settings. This review will summarize the different methods available for peripheral perfusion monitoring and will discuss the advantages and disadvantages of each approach.

## Introduction

Hemodynamic instability creates an imbalance between oxygen delivery and consumption and is an important contributor to organ failure (1). Hemodynamic monitoring is crucial to identify inadequate tissue perfusion in order to prevent organ dysfunction and death (2). Both global and peripheral biomarkers of tissue perfusion are used clinically as proxies for hemodynamic status. Global measurements often require invasive techniques, and the extent to which they reflect tissue oxygenation has been questioned (3).

Blood is diverted from less vital to more vital organs in response to circulatory failure (3–5). Compared to central organs, the peripheral, non-vital organs are the first to reflect hypoperfusion during shock and the last to reperfuse during resuscitation (6). As a result, clinicians shift from global to peripheral perfusion monitoring to promptly recognize deteriorating clinical status and to assess the effectiveness of resuscitation therapy (7). Measures of peripheral tissue perfusion have emerged as important tools to monitor the hemodynamic status of critically ill patients. Efforts have been made to determine the ideal method for assessing peripheral perfusion status, which should be non-invasive, rapid, reproducible, and easily measured.

Shock is the clinical manifestation of acute circulatory failure. It is characterized by signs of tissue hypoperfusion (8) and leads to impairments in cellular oxygen delivery (9, 10). Septic shock is a form of distributive shock that results from dysregulations in the host inflammatory response to infection (11). As a leading healthcare burden, septic shock accounts for one-third to one-half of all deaths occurring in hospital settings (12), with roughly 32 million sepsis cases per year (13). Given that peripheral perfusion status can be used to quickly assess shock severity (14), hemodynamic monitoring can allow clinicians to promptly initiate therapy, evaluate the effectiveness of interventions, and assess the patient throughout their recovery.

The monitoring of peripheral perfusion status is a central element to patient care. Methods to perform this measurement are of interest and can be complicated. Major modalities that are used to measure peripheral perfusion status include capillary refill time (CRT) and temperature. New technologies have been developed to calculate CRT. These devices measure either light reflected at the surface of the fingertip (reflected light), or light transmitted through the inside of the fingertip (transmitted light). These new approaches can enable clinicians to non-invasively monitor hemodynamic status with more accuracy than traditional measurements and may become a very valuable aspect of clinical medicine. This review will discuss the pros and cons of the available methods to monitor peripheral perfusion in critically ill patients. The available techniques will be grouped according to their mechanism of monitoring perfusion status, including reflected light, transmitted light, and temperature. Within these categories, the techniques will be further delineated as either subjective or objective methods of monitoring. This review therefore provides a novel schema for classifying the available methods of monitoring peripheral perfusion (Table 1).

**Table 1:** Summary of the available methods used to monitor peripheral perfusion.

**Method**	**Subjective measures**	**Objective measures**
Reflected light	Manual CRT (4, 15–17) – Quick, convenient, non-invasive, and inexpensive – Results in lower mortality and faster resolution of organ dysfunction than other resuscitation strategies (i.e., lactate) – Confounded by ambient/skin/core temperature, ambient lighting, gender, and age – Debatable interrater reliability	DCRT (18) – More accurate than clinical assessment – Limited in populations with darker skin
		Polarization spectroscopy (19) – Measurements correspond with the clinical definition of manual CRT – Time-consuming – Limited in chaotic clinical settings
		Novel device that adjusts for pressing strength/time (20) – Allows for standardized protocols – Limited in populations with darker skin
		Automated, pneumatic device (21) – Continuous measurements – May reduce clinician burden and inter-observer variability
		SDF (22–26) – Low cost, good portability, high sensitivity – Assesses deep sublingual arterioles – Time-consuming – Limited data utilization
Transmitted light	N/A	NIRS (27–30) – Non-invasive, easily monitored, reproducible – Time-consuming and expensive
		PPI (31–35) – Unambiguous, noninvasive, and continuous evaluations of perfusion status – Correlates with other variables of peripheral perfusion (pulse pressure, systolic blood pressure, calf blood-flow, oxygen delivery) – Predicts impending shock and mortality in septic patients status post-resuscitation – Limited in patients with hypothermia, embolism, or local vasospasm
		Q-CRT/BRT/CRI (14, 36–40) – Quick, non-invasive, and reproducible measurements – Predicts sepsis with same accuracy as lactate and qSOFA/SIRS scores – May promptly identify abnormal peripheral perfusion and allow for expedited treatment
Temperature	Clinical estimates of cool extremities (41–43) – Performed quickly and easily – Provides valuable insight on perfusion status – Large degree of inter-observer variability – May be confounded by ambient temperature	Body temperature gradients (4, 44, 45) – Greater accuracy and reproducibility than subjective assessments – Limited in anesthetized and cardiac surgical patients – May be confounded by ambient temperature

*CRT, capillary refill time; DCRT, digitally measured capillary refill time; SDF, sidestream dark field; NIRS, near infrared spectroscopy; PPI, peripheral perfusion index; Q-CRT, quantified CRT; BRT, blood refill time; CRI, capillary refill index; qSOFA, quick sequential organ failure assessment; SIRS, systemic inflammatory response syndrome*.

## Methods to Monitor Peripheral Perfusion

### Capillary Refill Methods

Capillary refill time is the time it takes for the color of a distal capillary bed to return to baseline after applying enough pressure to cause blanching. Delayed CRT is defined as >2 s (46) and indicates abnormal circulatory status (47). CRT is used clinically to assess peripheral circulation for signs of shock and dehydration (21).

### Reflected Light and Surface Color Changes

#### Subjective Measures

Clinicians routinely measure CRT using the naked eye. The value of these measurements in hemodynamic monitoring has been widely studied. Ait-Oufella et al. (15) examined septic shock patients 6 h after resuscitation. The investigators found that prolonged CRT was a strong predictive factor of 14-day mortality. Hernandez et al. (4) studied a mixed severe sepsis/septic shock population 6 h after resuscitation. The authors found that CRT <4 s was associated with correction of hyperlactatemia at 24 h. van Genderen et al. (16) examined the diagnostic accuracy of different peripheral perfusion measures in identifying surgical patients at high risk of developing post-operative complications. The investigators reported that CRT displayed the highest diagnostic accuracy [area under the curve (AUC) 0.91] and was independent of systemic hemodynamics. Hernandez et al. (17) compared organ dysfunction in septic shock patients 72 h after different methods of resuscitation. The authors reported that peripheral perfusion–targeted resuscitation was associated with less organ dysfunction compared to lactate-targeted resuscitation [mean sequential organ failure assessment (SOFA) score 5.6 vs. 6.6]. Peripheral perfusion–targeted resuscitation also trended toward reduced 28-day mortality, but did not reach the proposed statistical significance threshold (*p* = 0.06). Zampieri et al. (48) reassessed the results of this trial using both a *post-hoc* Bayesian analysis and a mixed logistic regression analysis. The authors reported a very high probability that peripheral perfusion–targeted resuscitation results in lower mortality and faster resolution of organ dysfunction than lactate-targeted resuscitation strategies. These findings highlight the prognostic value and therapeutic potential of the manual CRT-test.

Subjective CRT measurements are quick, convenient, non-invasive, and inexpensive methods of measuring hemodynamic status. However, there are important limitations. Measurements can be confounded by ambient (49–51), skin (49, 52, 53), and core temperature (50), ambient lighting (54), gender and age (50–52). Clinicians may interpret surface color changes differently, which raises concerns about the reliability of the CRT-test. Ait-Oufella et al. (15) found that CRT is very reproducible with an excellent inter-rater concordance (80%: index finger; 95%: knee). van Genderen et al. (16) reported a good overall agreement for inter-rater reliability of CRT between different examiners on different post-operative days (κ = 0.74–0.91). However, Alsma et al. (55) reported that inter-observer agreement on CRT is moderate at best, and higher for the distal phalanx (κ = 0.40) than for the sternum (κ = 0.30). Brabrand et al. (56) found only moderate inter-observer reliability (κ = 0.56) when observers categorized CRT as normal or abnormal. When CRT was measured in seconds, the investigators found an acceptable interclass correlation (ICC) of 0.62. Quantitative measures of CRT with clearly defined cut-offs appear to reduce discrepancies between observers (57).

It is possible that training may improve the reproducibility of the CRT-test. In our laboratory, we analyzed CRT measurements made by observers with varying training levels (58). We found that the mean intra-observer reliability was higher in clinicians than non-clinicians (0.46 vs. 0.25). It was also the highest in attending physicians and physician assistants, followed by residents, nurses, and non-clinicians. Standardization of compression strength may also improve reproducibility. Ait-Oufella et al. (15) reported excellent inter-rater concordance using 15 s of firm pressure. Alsma et al. (55) found only slightly higher inter-observer correlation using 15 s of pressure compared to 5 s. Considering practicality in emergent settings, investigators recommend the use of 5 s of moderate, firm pressure to perform CRT measurements (55, 57). Standardization of the protocol may help overcome the shortcomings in routinely measuring CRT.

#### Objective Measures

Technology has been developed to objectively calculate CRT. These devices also measure reflected light, but eliminate the variability that exists when clinicians manually measure CRT. Shavit et al. (18) introduced the concept of digitally measured CRT (DCRT). DCRT is calculated as the time between the release of fingertip compression and the recovery frame. In children with gastroenteritis, DCRT was more accurate at assessing the presence of significant dehydration than overall clinical assessment by experienced pediatric ED physicians (AUC 0.99 vs. 0.88). Kawaguchi et al. (20) developed a device that adjusts for pressing strength and time to determine the characteristics of the optimal fingernail compression. The authors reported that fingernail compressions <2 s resulted in unreliable CRT measurements. They found significant differences in CRT at pressing strengths of 1 newton (N) and 3 N, but no significant differences between 3, 5, and 7 N. The investigators therefore recommended compressions using 3–7 N of pressure for 2 s. Development of devices that uniformly apply these compression settings may improve the precision of hemodynamic monitoring in clinical settings. Using an automated, pneumatic device, Blaxter et al. (21) reported a statistically significant increase in CRT in the majority of patients who underwent forearm cooling. As the device provides continuous measurements, it can repeatedly monitor hemodynamic status while reducing clinician burden and inter-observer variability. John et al. (19) used video mode polarization spectroscopy to quantify changes in red blood cell (RBC) concentration. The authors found that tRtB1 (rapid return of RBC concentration to baseline after release of fingertip pressure) corresponds best with the clinical definition of visually inspected CRT. However, clinicians may actually measure t_pk_ (onset of hyperemia after resolution of blanching) when they perform the test. The naked eye alone may therefore be incapable of capturing the fundamentals of the CRT test. Implementation of this software into clinical care may allow clinicians to monitor perfusion status and guide clinical decision-making with more accuracy. However, this technology is limited in chaotic clinical settings, where recorded video data can be shaky and unfocused (36). In contrast to fingertip assessments, certain technologies evaluate perfusion status via analysis of reflected light at different areas of the peripheral surface. Investigators have used sidestream dark-field (SDF) imaging (22, 23) to assess peripheral perfusion via evaluation of the sublingual microcirculation. In critically ill patients, Klijn et al. (24) reported that SDF assessed tissue perfusion and oxygenation was not inferior to invasive hemodynamic measurements in monitoring fluid responsiveness. SDF provides clear capillary imaging and can evaluate deep sublingual arterioles (22, 25). However, a large amount of data is discarded due to image quality artifacts and manual tracing of the vessels is too time consuming to be practical for clinical use. The development of automated devices would increase the clinical utility of SDF measurements (26).

Objective CRT measurements provide detailed data, improve reproducibility, and minimize observer bias. There are also disadvantages. Technology that assesses skin color changes (18, 20) is limited in populations with darker skin. The current design of these devices is impractical for routine use in clinical settings (18) and the procedures are time-consuming (19). Future research should focus on making adjustments that reduce procedural time and allow these devices to be easily implemented into patient care.

### Transmitted Light and Spectrophotometric Methods

New technologies measure peripheral perfusion by analyzing light transmitted through the inside of the fingertip. Since visual assessments cannot be performed, there are no subjective measures of peripheral perfusion using this methodology.

Near infrared spectroscopy (NIRS) analyzes spectra in the near-infrared range to quantify oxyhemoglobin and deoxyhemoglobin levels in order to assess peripheral tissue oxygen saturation (StO_2_) (27, 28). The utility of NIRS for monitoring critically ill patients remains uncertain (28). Lima et al. (29) investigated the relationship between thenar StO_2_ during a vascular occlusion test (VOT) to the peripheral perfusion status and clinical outcome of critically ill patients. The authors reported a significantly lower baseline StO_2_ and StO_2_ recovery rate in patients with abnormal peripheral perfusion compared to patients with normal peripheral perfusion (72 vs. 81 and 1.9 vs. 3.2, respectively). These findings were independent of disease condition and hemodynamic status. In a follow-up study, Lima et al. (28) investigated the effect of peripheral vasoconstriction on thenar StO_2_. After body surface cooling, the authors reported a significant decrease in StO_2_ (82–72%) and StO_2_ recovery rate (3.0–1.7%/s). Together, these findings suggest that peripheral tissue oxygenation varies according to peripheral circulation status. StO_2_ measurements should therefore be interpreted in the context of other markers of peripheral circulation, such as skin temperature. Given that perfusion status continually changes in critically ill patients, clinicians must carefully consider peripheral circulatory status when using NIRS for hemodynamic monitoring. Although NIRS can non-invasively evaluate perfusion, it is limited by the fact that the measurements are time consuming and expensive (30).

Peripheral perfusion index (PPI) has been investigated for its use in hemodynamic monitoring. Using pulse oximetry, PPI is calculated from the ratio between the pulsatile and non-pulsatile signals of absorbed light (31) and provides insight on the circulatory status of vital organs during shock (32). In a study on healthy newborn infants, Zaramella et al. (32) compared the relationship between foot PPI and variables of peripheral perfusion measured by NIRS on the calf. The authors reported a significant correlation between foot PPI and both calf blood flow (*r* = 0.32) and oxygen delivery (*r* = 0.32). In a study on 100 children undergoing hemodynamic monitoring, Sivaprasath et al. (33) reported that PPI had a good correlation with pulse pressure and systolic blood pressure in all age groups, and a weak correlation with mean arterial blood pressure and diastolic blood pressure. The authors concluded that a 57% reduction in PPI from baseline may predict impending shock in children. He at al. (34) explored the prognostic value of PPI in septic patients. The authors reported that PPI was significantly correlated with baseline transcutaneous carbon dioxide tensions (PtcO_2_), 10 min-oxygen challenge test (OCT) and oxygen challenge index (OCI). The authors also found significantly lower PPI, 10 min-OCT, and OCI values in non-survivors compared to survivors. These variables predicted ICU mortality with similar accuracy to arterial lactate level. PPI therefore appears to be a simple yet powerful predictor of mortality in septic patients status post-resuscitation. The results of these studies support the use of PPI for unambiguous, non-invasive, and continuous evaluations of global resuscitation status and outcome. However, PPI is limited in patients with hypothermia, embolism, or local vasospasm (35). Additional studies are necessary to support the routine monitoring of PPI as a parameter to detect impending shock and improve clinical outcomes.

Recent research has focused on the development of new technology that quantifies CRT using a pulse oximeter, which investigators have called quantified CRT (Q-CRT). Morimura et al. (14) first introduced this method. The authors analyzed the infrared transmitted light intensity (TLI) emitted from a pulse oximeter senor. They defined Q-CRT as the time in seconds from the release of fingertip compression to TLI reaching 90% of baseline. The authors reported that Q-CRT was significantly correlated with blood lactate levels in ICU patients (*r*_*s*_ = 0.681). Q-CRT has also been correlated with venous blood lactate levels in ED patients (37). Together, these results suggest that Q-CRT might be an effective measure of insufficient global tissue perfusion and shock in both the ICU and ED. In ED patients with suspected infection, Yasufumi et al. (38) investigated the ability of Q-CRT to predict sepsis compared with quick sequential organ failure assessment (qSOFA) and systemic inflammatory response syndrome (SIRS) scores. The authors reported that the accuracy of Q-CRT in predicting sepsis was comparable to qSOFA scores, SIRS scores, and lactate level. Q-CRT may therefore be a quicker and non-invasive alternative to evaluate patients with suspected sepsis.

**Figure 1:**
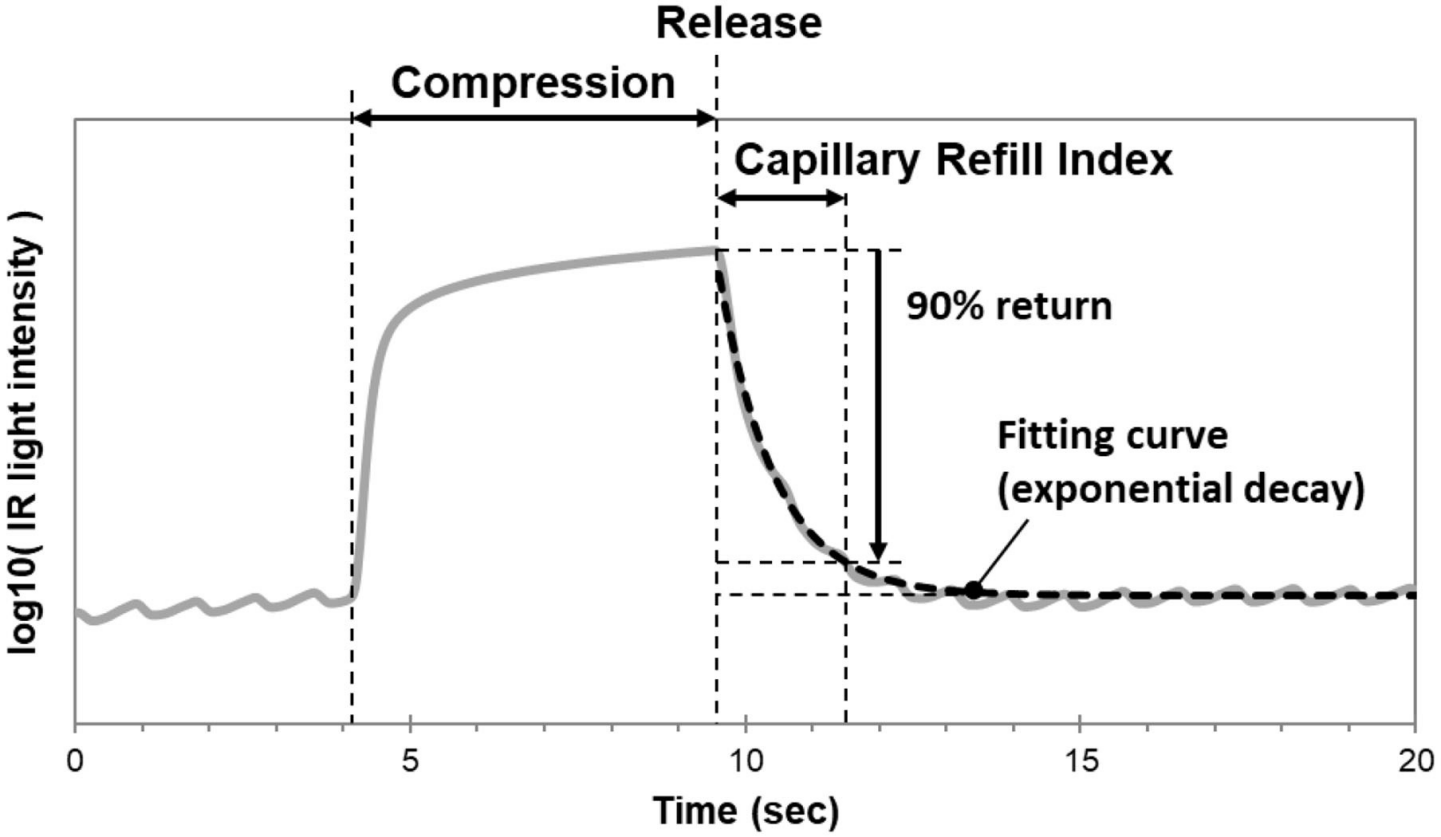
Mechanism of the CRI monitoring device. The curve fitting the recovery phase of the TLI waveform is modeled as an exponential decay using the least squares method. CRI is measured as the time at which the fitting curve returns to 90% of baseline.

In our laboratory, we modified the TLI calculation used to measure Q-CRT. We modeled the curve fitting the recovery phase of the TLI waveform as an exponential decay using the least squares method, and measured the time at which the fitting curve returned to 90% (Figure 1). This improved measurement was named blood refill time (BRT) (39, 40) and later referred to as capillary refill index (CRI) (36). In a healthy volunteer (39), we found that our device successfully detected prolonged BRT (5.8 s) after fingertip cooling to 22.8°C. In 30 healthy volunteers (40), we measured BRT at room temperature, after immersion in cold water, and after re-warming by warm water. We reported that the “cold” group had significantly longer BRT (4.67 s) than the “room temperature” (1.96 s) and “re-warm” groups (1.96 s). Our data suggests a causal relationship between temperature and peripheral blood perfusion. Healthcare providers routinely encounter patients with cool fingertips when performing bedside evaluations of CRT. Our results suggest that clinicians should interpret these measurements with caution when performed in the setting of unknown fingertip temperature.

We compared the accuracy of CRI to CRT calculated via software analysis of recorded fingertip compression videos (36). We measured CRT and CRI at room temperature, after immersion in cold water, and after re-warming by warm water. To avoid procedural variability in compression, the fingertips were compressed pneumatically for both CRI and CRT at the same pressure and duration. We found that there was a strong correlation between CRI and CRT (*r* = 0.89). We performed a validation study in the ED to clinically evaluate the accuracy of our device (36). We reported a strong correlation between CRI and CRT (*r* = 0.76). Given the use of software analysis, we believe our study provides reliable evidence that the CRI algorithm is representative of the CRT measurements performed in clinical practice. We also reported higher CRI and CRT in ED patients compared to healthy volunteers at room temperature. Using a Bland-Altman analysis, we found that CRI was consistently higher than CRT (difference = +1.01). Although CRI and CRT measurements both represent peripheral perfusion status, this data suggests that the absolute value of the measurements may not be equal. Because CRI was associated with a systematic bias rather than random errors, we recommend it as a reliable and objective alternative to the manual CRT-test.

Q-CRT/CRI minimizes observer variability and provides immediate and reproducible data regarding the circulatory status of critically ill patients. Given that resuscitation strategies rooted in peripheral perfusion monitoring result in lower mortality, faster resolution of organ dysfunction, and decreased fluid requirements (17, 48), Q-CRT/CRI may have tremendous clinical potential. However, future research is necessary in populations of critically ill patients in order to evaluate the efficacy of Q-CRT/CRI in lowering mortality and reducing individual requirements (i.e., vasopressor, mechanical ventilation, and renal replacement therapies). With further clinical support, this technology may be a promising alternative for continuous monitoring and spot check measurements of peripheral perfusion.

## Temperature

Body temperature is distributed both centrally (body core) and peripherally (body shell and environment) (59). Thermoregulatory status provides insight about the clinical condition of patients in intensive/critical care units. In response to shock, blood flow is restricted to central/vital organs at the expense of peripheral organs. Peripheral temperature is therefore used as an indicator of hemodynamic status (16, 60). Septic shock is divided into two categories: “cold” and “warm” shock (41, 61). Some authors believe that this distinction might confuse the interpretation of perfusion state (62). Regardless, studies show that peripheral temperature is still useful in differentiating well-perfused from hypo-perfused patients (63).

### Subjective Measures (Clinical Estimates)

Healthcare providers use clinical judgment to estimate peripheral body temperature in order to quickly evaluate hemodynamic status. In ICU patients, Kaplan et al. (42) compared clinician assessment of distal extremity skin temperature (warm or cool) to objective markers of hypoperfusion. The authors found that patients with cool extremities had significantly higher serum lactate levels and lower cardiac index compared to patients with warm skin temperature. Hasdai et al. (43) reported that the presence of cold and clammy skin was an independent predictor of 30-day mortality in cardiogenic shock patients. 48 h after resuscitation, Lima et al. (41) found that ICU patients with cool extremities had significantly higher rates of organ failure compared to patients with normal skin temperature (SOFA score 9 vs. 7).

Peripheral temperature measurements can be performed quickly and easily while providing valuable insight on perfusion status. However, there is a large degree of variability, since what is considered “cool” to one clinician may not always be consistent. Changes in ambient temperature may also affect clinical estimates of skin temperature (41).

### Objective Measures (Temperature Gradients)

Body temperature gradients provide objective measures of peripheral perfusion status. The gradients are created by calculating the temperature difference between two points, such as central-to-toe (Tc-toe), forearm-to-fingertip (Tskin-diff), and peripheral-to-ambient. Skin temperature can be measured using infrared thermometer (59), thermocouple disposable probes (64), or infrared thermography (65). Regardless of the modality used, these gradients provide non-invasive, accurate measures of thermoregulatory peripheral vasoconstriction (66). In response to shock, there is a reduction in fingertip blood flow in order to maintain perfusion of vital organs. This causes Tskin-diff and Tc-toe gradients to increase in the presence of constant environmental conditions (60). Peripheral-to-ambient gradients decrease during shock (3), despite some limitations that exist from this interpretation and from using ambient temperature as a marker.

In critically ill patients, Joly et al. (44) reported a significantly lower toe-to-ambient gradient in non-survivors (0.9°C) than in survivors (3.4°C). In septic patients, Hernandez et al. (4) found that return of Tc-toe to normal within the first 6 h of resuscitation was independently associated with successful resuscitation. It was also predictive of hyperlactatemia normalization at 24 h. In septic patients, Bourcier et al. (45) reported significant decreases in toe-to-room temperature gradients in patients who died from multiple organ failure (−0.2°C) compared to survivors (+3.9°C). Toe-to-room temperature gradient was also significantly correlated with other measures of tissue perfusion, including urine output, arterial lactate level, knee CRT, and mottling score.

Body temperature gradients provide better reproducibility than clinical estimates of cool hands/feet (67) and are more accurate reflections of peripheral blood flow than skin temperature alone (3, 68, 69). Abnormal gradients can be used as early indicators of abnormal perfusion, while therapeutic efficacy can be monitored by normalization of the gradient (70). As most critically ill adult patients undergo invasive monitoring, the use of body temperature gradients provides a non-invasive, economic, and effective alternative to monitor circulatory status (59). However, body temperature gradients are limited in certain populations, including anesthetized (71) and cardiac surgical patients (72). Differences in ambient temperature between the two measurement sites may also influence Tc-toe and Tskin-diff gradients. However, any fluctuations in ambient temperature should affect both sites similarly and minimize any potential confounds (41).

## Recommendations and Conclusions

In order to promptly initiate life-saving clinical interventions and improve outcomes, an early recognition of shock is key. Many authors therefore recommend peripheral perfusion measures to continuously assess the hemodynamic status of critically ill patients. However, current sepsis bundles suggest the need for the reassessment of resuscitation guidelines. It appears that the use of therapies guided by peripheral perfusion measurements result in favorable clinical outcomes. It is important to acknowledge that our review does not discuss all of the available technologies that may be used to evaluate peripheral perfusion status, such as laser doppler flowmetry (LDF), infrared thermography (73), and PulseCam technology (74). Nevertheless, clinicians should choose among the available techniques with an understanding of the pros and cons of each approach (Table 1). Newer technologies that measure CRT, such as the objective CRI device used in our laboratory, meet many of the important criteria of assessing peripheral perfusion and are promising tools to monitor shock status at the bedside. Future studies focused on peripheral perfusion should further define the clinical implications of these devices, including their utility in modulating response to treatment. Adjustments should also be made to make the devices more practical for routine clinical use. The ability of these technologies to provide uniform/reproducible measurements across different clinical settings, decrease the use of hospital resources, and improve clinical outcomes would strengthen the role of peripheral perfusion monitoring in the bedside evaluation of hemodynamic status.

## Author Contributions

KSh and JF designed the conception of the work. JF drafted. KSh and KSa edited the manuscript. LB supervised and enabled the work. All authors added intellectual content, interpretations of the work, critically revised the paper, and gave final approval of the version to be published.

## Conflict of Interest

KSa is an employee of Nihon Kohden Corporation and Nihon Kohden Innovation Center, Inc. A pulse oximeter with the CRI function is marketed in Japan. This does not alter the authors' adherence to all the journal's policies on sharing data and materials. KSh and LB had a patent right of metabolic measurements in critically ill patients. KSh had a grant/research support from Nihon Kohden Corp. LB had a grant/research support from Philips Healthcare, the NIH, Nihon Kohden Corp., Zoll Medical Corp., PCORI, BrainCool, and United Therapeutics and owes patents including seven issued patents and several pending patents involving the use of medical slurries as human coolant devices to create slurries, reperfusion cocktails, and measurement of respiratory quotient. The remaining author declares that the research was conducted in the absence of any commercial or financial relationships that could be construed as a potential conflict of interest.

## Abbreviations

CRT: Capillary refill time
AUC: area under the curve
SOFA: sequential organ failure assessment
ICC: interclass correlation
RBC: red blood cell
DCRT: digitally measured capillary refill time
N: newton
SDF: sidestream dark field
NIRS: near infrared spectroscopy
PPI: peripheral perfusion index
PtcO_2_: transcutaneous carbon dioxide tensions
OCT: 10 min-oxygen challenge test
OCI: oxygen challenge index
Q-CRT: quantified CRT
qSOFA: quick sequential organ failure assessment
SIRS: systemic inflammatory response syndrome
BRT: blood refill time
CRI: capillary refill index
StO_2_: peripheral tissue oxygen saturation
VOT: vascular occlusion test
TLI: total light intensity
Tc-toe: central-to-toe
Tskin-diff: forearm-to-fingertip
LDF: laser doppler flowmetry.
